# The PFNA in treatment of intertrochanteric fractures with or without lateral wall fracture in elderly patients: a retrospective cohort study

**DOI:** 10.1186/s40001-023-01332-y

**Published:** 2023-09-27

**Authors:** Yunfeng Tang, Dong Wang, Limin Wang, Wei Xiong, Qian Fang, Wei Lin, Guanglin Wang

**Affiliations:** 1 Trauma Medical Center, Department of Orthopaedic Surgery, West China Hospital, West China Medical School, Sichuan University, No. 37 Guoxue Road, Chengdu, 610041 Sichuan People’s Republic of China; 2 West China Women’s and Children’s Hospital, No. 17 People’s South Road, Chengdu, Sichuan China

**Keywords:** Intertrochanteric fractures, Lateral wall, Proximal femoral nail antirotation

## Abstract

**Background:**

There is no consensus about intertrochanteric fractures with lateral wall treated with intramedullary nail—proximal femoral nail antirotation (PFNA). The aim of the present study was to compare function outcomes between lateral wall and no lateral wall fractures after surgery by PFNA.

**Methods:**

This retrospective study evaluated patients with or without lateral wall fractures who underwent PFNA between January 2015 and June 2018. The operative time, intraoperative blood loss, time to fracture healing, complications and functional outcomes qualified by Harris hip score and Parker − Palmer mobility score (PPMS) were compared between the two groups.

**Results:**

Two groups were comparable with regard to patient age, sexual distribution, mechanism of injury, fracture type, body mass index (BMI), Time to surgery, American Society of Anesthesiologists (ASA) score and quality of reduction. The incomplete group had a longer operation time (54.1 ± 8.74 min vs. 51.0 ± 9.86 min) and more intraoperative blood loss (228.4 ± 48.8 ml vs. 151.3 ± 43.5 ml) in comparison with the control group (*P* < 0.01). Regarding functional outcome, the HHSs of the two groups were 76.2 ± 11.6 vs 75.6 ± 12.5 at the 3 months (*P* = 0.603), 81.9 ± 9.4 vs 82.6 ± 8.7 at the six months (*P* = 0.224), 83.8 ± 6.6 vs 84.5 ± 6.0 at the twelve months 85.2 ± 5.5 vs 86.0 ± 5.8 at the twenty-four months (*P* > 0.05), respectively. Similar results were obtained about PPMS. We found no difference in Weight bearing time, Time of fracture healing, and Complications between incomplete group and intact group.

**Conclusions:**

There is no substantial difference in functional results or complication rates for intertrochanteric fractures with lateral wall fractures, except from increased blood loss and operation time. We believe that an intramedullary nail will be sufficient to repair an intertrochanteric fracture with or without a lateral wall fracture.

## Background

Hip fractures are one of the most common injuries in the elderly, who often suffer from other serious health problems. According to the latest epidemiological survey, the incidence rates of intertrochanteric fractures were 171/100,000 and have brought huge burden to the health care systems and society [1, 2]. Some researchers hypothesized that when intertrochanteric fractures involve the lateral wall of the femur, defined anatomically as the lateral cortex of the distal femoral shaft, also known as the “calcar”, they are not adequately treated with sliding hip screws [3, 4]. Gotfried Y first emphasized the importance of lateral trochanteric wall and certainly suggested that an intact lateral trochanteric wall played a key role in the stabilization of unstable fractures [5]. Im and Palm et al. have successively verified the role of the lateral wall, proposing that the anatomical lateral wall refers to the proximal lateral cortex of the femur that extends up to the lateral femoral crest, connects to the greater trochanter, and down to the midpoint plane of the lesser trochanter [6, 7]. The intact lateral wall takes about 67% of all intertrochanteric fractures, and incompetent lateral wall takes 33% [8].Lateral wall provides the best opportunity for osteosynthesis with the proximal part of the fracture complex. If this fracture of lateral wall was ignored, a pertrochanteric fracture may convert into a subtrochanteric fracture, which cause a more severe problem. Intramedullary fixation could effectively prevent lateral excessive sliding of proximal fragment and medialization of the shaft and has shorter operation time, faster full weight bearing, lower incidence of failure of the plant compared with extramedullary fixation [9, 10]. Duration of the operation, intact lateral wall demonstrates the simple fracture lines, and it is easy to reduce. In contrast, incompetent lateral wall is usually difficult to achieve good reduction because of multifragmentary fractures. There are a few of studies involving the quality of reduction and prognosis affected by the lateral wall. Studies have shown that the fixation failure complication rates were only 1.2 ~ 3.9% in intertrochanteric fracture treated with intramedullary fixation [11, 12]. However, when subgroup analysis of unstable intertrochanteric fractures was performed, the fixation failure complication rates of intramedullary nail technique were up to 20.5 ~ 24.2% [13, 14]. Currently, there is a lack of long term follow-up study on the impact of lateral wall on reduction quality and prognosis in the treatment of intramedullary nails. Thus, the present study is aimed at evaluating the effect of the integrity of lateral wall on the quality of reduction and outcome in intertrochanteric fracture treated with intramedullary nails after long-term follow-up.

## Methods

This was a retrospective observational study, approved by the ethics committee of The West China Hospital (Approval Number: 20211115). Written informed consent was obtained from each participant before its commencement. Between January 2015 and June 2018, 158 consecutive patients with intertrochanteric fractures treated with closed reduction with the intramedullary fixation by PFNA were included for data analysis.

### Inclusion and exclusion criteria

The inclusion criteria were (1) age ≥ 60 years; (2) unilateral intertrochanteric fractures were confirmed using radiography, and the integrity of lateral wall could be distinguished; (3) operative treatment of closed reduction and internal fixation was undergone by PFNA; and (4) at least 12 months of follow-up.

**Fig. 1:**
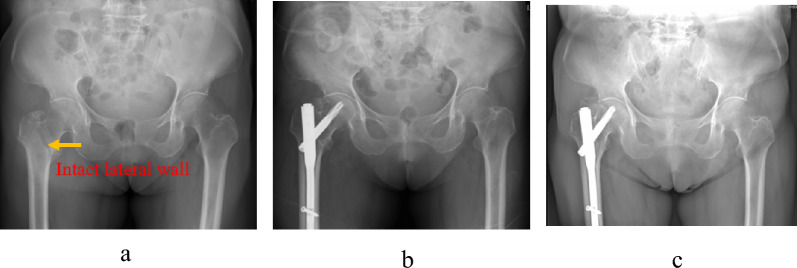
A 67-year-old female was treated for a right intertrochanteric fracture without lateral wall fracture (AO type A1.1) via closed reduction and internal fixation. **a** Radiograph of right hip showing intertrochanteric fracture without lateral wall fracture. **b** immediate postoperative radiograph showing sound reduction and after intramedullary nail. **c** three years follow-up radiograph showing bone union and no varus deformity

**Fig. 2:**
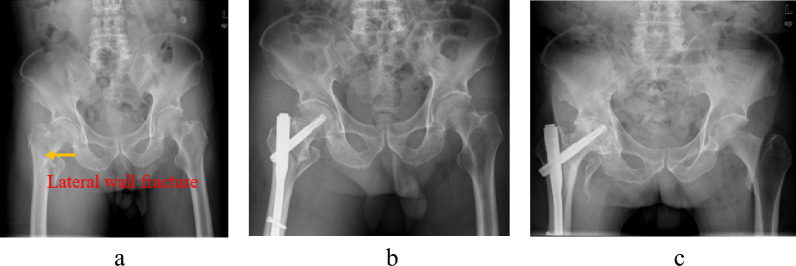
A 72-year-old female was treated for a right intertrochanteric fracture with lateral wall fracture (AO type A2.2) via closed reduction and internal fixation. **a** Radiograph of right hip showing intertrochanteric fracture with lateral wall fracture. **b** immediate postoperative radiograph after intramedullary nail. **c** Five years follow-up radiograph showing bone union and hip joint arthritis

The exclusion criteria were (1) age < 60 years, (2) multiple injuries with intertrochanteric fractures and other fractures and only operation for intertrochanteric fractures, (3) ASA score V and (4) patient refusal to participate. We searched the medical system records for patients with intertrochanteric fractures (Figs. 1, 2).

### Surgical strategy

Patients were placed supine on the operating table, with the lower limb in traction. A preoperative fluoroscopy in anterior − posterior and axial views was obtained to evaluate fracture alignment after the reduction maneuvers. The reduction was judged satisfactory when the main fracture fragments were correctly aligned in both fluoroscopic views. Then an approximately 4- to 7-cm proximal and longitudinal incision was made through the fascia and gluteus to expose the tip of the greater trochanter. The proximal canal was then opened by evenly applied force to avoid breakage of the greater trochanter. After insertion of a reamed nail, fluoroscopy was performed to evaluate the fracture situation. By the anteroposterior C-arm fluoroscopy, the guide pin is located in 1/3 of the femoral neck and located central of the femoral neck by lateral fluoroscopy. If the position of the guide pin was poor, then the pin should be adjusted to the correct position, but repeated adjustments should be avoided.

### Grouping methods

The patients were divided into 2 groups, according to integrity of lateral wall. When the thickness of lateral wall was less than 20.5 mm, it was divided into incompetent group; when it was longer than 20.5 mm, it was divided into intact group [15]. Finally, 72 patients are without lateral fracture and 86 patients are with lateral fractures.

### Outcome measurement

Baseline data were recorded in the two groups, including age, gender, injury type, BMI, injury side, tip − apex distance (TAD), and quality of reduction. Operation time, intraoperative blood loss, time of fracture healing, full weight-bearing time and postoperative fixation failure between the two groups were assessed in this study. At the finally postoperative follow-up, Harris hip score [16] the Parker − Palmer mobility score [17] were performed to evaluate the functional states and mobility. complications included loss of reduction, cutout, implant breakage, malunion, nonunion, deep vein thrombosis and wound infection, which were recorded during the follow-up. Malunion was defined as less than 50% contact between the proximal and distal fragments or collodiaphyseal angle of less than 120°. Bone union was defined by the following radiographic parameters: restoration of cortical continuity, loss of a clear fracture line, and presence of callus. Nonunion which means nonunion of the fracture itself, not that of the third fragment, was defined as the state in which disturbed consolidation of a fracture that needs further surgical intervention or a prolonged healing time of more than 12 months or more TAD described by Baumgaertner et al. was calculated by using the anteroposterior and lateral radiographs to evaluate the implant position [18]. The quality of postoperative reduction was graded as good, acceptable, or poor [13, 19] (Table 1).

**Table 1:** Preoperative baseline characteristics

	Incomplete lateral wall	Intact lateral wall	*P* value
Total number of patients	72	86	**–**
Male/female	27/45	36/50	0.577
Age, years	78.07 ± 7.56	79.61 ± 7.44	0.623
BMI, kg/m^2^	21.22 ± 1.80	21.58 ± 1.95	0.353
ASA class, *n*			0.923
1	12	15	
2	23	31	
3	34	36	
4	3	4	
Side, right/left	34/38	37/49	0,597
Time to surgery			0.635
< 24 h	47	53	
> 24 h	25	33	
TAD			
> 25 mm	41	53	
< 25 mm	31	33	
Anesthesia			0.421
General	52	57	
Spinal	20	29	
Comorbidities			0.840
Hypertension	20	25	
Diabetes	15	21	
Cerebral infarction	5	3	
Urinary tract infection	7	6	
Stroke	3	2	
Without Preoperative comorbidities	22	29	
Trauma mechanism High-energy	13	24	0.145
Low energy	59	62	
Quality of reduction			0.900
Good	46	54	
Acceptable	20	23	
Unacceptable	6	9	

*BMI* body mass index; *ASA* American Society of Anesthesiologists

### Statistical analysis

Statistical analysis was performed using SPSS version 26.0 (SPSS Inc., Chicago, IL, USA). We assessed whether measurement data were normally distributed using the Shapiro − Wilk test and then using independent-samples t tests. For frequency data, the chi square or Fisher’s exact test was used. If *p* < 0.05, there was the considered statistically significant difference.

## Results

Two groups were comparable with regard to patient age, sexual distribution, mechanism of injury, fracture type, BMI, Time to surgery, ASA score and quality of reduction. The incomplete group had a longer operation time (54.1 ± 8.74 min vs. 51.0 ± 9.86 min) and more intraoperative blood loss (228.4 ± 48.8 ml vs. 151.3 ± 43.5 ml) in comparison with the control group (*P* < 0.01). Regarding functional outcome, the HHSs of the two groups were 76.2 ± 11.6 vs 75.6 ± 12.5 at the three months (*P* = 0.603), 81.9 ± 9.4 vs 82.6 ± 8.7 at the six months (*P* = 0.224), 83.8 ± 6.6 vs 84.5 ± 6.0 at the twelve months 85.2 ± 5.5 vs 86.0 ± 5.8 at the twenty-four months (*P* > 0.05), respectively. Similar results were obtained about PPMS. We found no difference in Weight bearing time, Time of fracture healing, and Complications between incomplete group and intact group. Table 2

**Table 2:** Perioperative and postoperative follow-up data

	Incomplete lateral wall	Intact lateral wall	*P* value
Operation time (min)	54.1 ± 8.74	51.0 ± 9.86	0.037
Intraoperative blood loss(ml)	228.4 ± 48.8	151.3 ± 43.5	0.001
Follow-up time (years)	4.16 ± 0.62	4.11 ± 0.63	0.558
Weight bearing time (months)	3.42 ± 0.65	3.26 ± 0.55	0.126
Time of fracture healing	3.96 ± 0.70	3.68 ± 0.64	0.112
Complications			
Postoperative fixation failure	2	1	0.592
Loss of reduction	1	0	0.456
Cutout	1	0	0.456
Implant breakage	1	2	0.592
Malunion	3	0	0.092
Nonunion	2	2	1.000
Deep vein thrombosis	4	7	0.542
Wound infection	0	0	–
HHS			
3 months	75.6 ± 12.5	76.2 ± 11.6	0.605
6 months	81.9 ± 9.4	82.6 ± 8.7	0.224
12 months	83.8 ± 6.6	84.5 ± 6.0	0.154
24 months	85.2 ± 5.5	86.0 ± 5.8	0.125
PPMS			
6 months	6.7 ± 2.1	7.0 ± 1.8	0.356
12 months	7.7 ± 1.3	7.9 ± 1.1	0.145
24 months	8.2 ± 1.1	8.3 ± 1.0	0.112

*HHS* Harris Hip Score, *PPMS* Parker − Palmer mobility score

## Discussion

Lateral wall is an important structure in the stabilization of intertrochanteric femur fracture, which can provide a lateral buttress for the proximal fragment. Therefore, the lateral wall fracture can result in proximal femur collapse, which causes postoperative morbidity, significant fixation failure rates, and even a bad prognosis. [5, 20]. According to the relative study, the fracture of the lateral trochanteric wall was the primary independent predictor of fixation failure complication requiring reoperation. [7]. A multivariate regression analysis found that lateral wall fracture was correlated to fixation cutout. [21]. Therefore, the reduction of lateral wall damages needs more attention in unstable intertrochanteric femoral fractures. The study was initiated to compare the outcomes of intertrochanteric fracture with and without lateral wall fractures.

Internal fixation is complicated by an intertrochanteric fracture with lateral wall fractures. Forces are carried from the femoral head to the femoral shaft when the hip joint is loaded, most notably via the posteromedial cortex. Stable fractures are those in which the posteromedial cortex and calcar femoral remain intact. When the hip is loaded, unstable fractures tend to outward rotate and Varus angulate, resulting in limb shortening and abductor mechanism insufficiency [5]. In the surgical treatment of stable intertrochanteric fractures, sliding and compression dynamic hip screws are considered the “gold standard” [22, 23]. In intertrochanteric fractures treated with sliding screw plates, Wolfgang et al. observed mechanical complication rates of 9% for stable fractures and 19% for unstable fractures [24]. Cutout of the neck screw in the femoral head is the most common cause of fixation failure [24]. Palm et al. thought that a sliding compression hip screw was not sufficient for treatment of fractures involving the lateral wall and more methods should be needed to manage this condition [7]. However, Hu et al. thought that anatomic locking plate could be used for intertrochanteric fractures with lateral wall crack especially for severe comminuted fractures, difficult for intramedullary nailing to avoid reinjury of lateral wall [25]. Using sliding hip screw in fractures with broken lateral wall could result in collapse, limb length shortening and poorer functional outcome [26, 27]. Gupta RK et al. showed that lateral wall reconstruction using a trochanteric stable plate in combination with a dynamic hip screw can be successful proximal femoral nail antirotation and locking compression plate have good effectiveness in the treatment of intertrochanteric fractures with the lateral unsubstantial femoral wall in the elderly patients [28]. However, Haq RU et al. found that proximal femoral nail was a better implant than reverse distal femoral locking compression plate for intertrochanteric fractures with compromised lateral wall because of favorable intraoperative variables, better functional outcome and lower failure rates. Currently, because of fewer implant failures and high union rates, there is a trend toward more frequent use of intramedullary fixation of intertrochanteric fractures [2, 10, 29]. Clinical and biomechanical study show that anatomic reduction is the key to success in the management of fractures of the proximal femur [30, 31]. Rao et al. got a conclusion that the benefits of the anatomic reduction are that weight bearing can be commenced early, the machine can be used for stable and unstable intertrochanteric fractures with same technique, and fixation is inflexible and lets in for compression of the fracture site, while preserving alignment [30]. Chang et al. indicates that anatomic reduction of four-part intertrochanteric fractures with the sliding hip screw provides significantly higher compression across the calcar region and significantly lower tensile strain on the plate than fractures reduced by medial displacement osteotomy [31]. However, Deng et al. have got a conclusion in a controlled study that there was no significant difference in complications between intact and incomplete lateral wall fractures [32]. In this study, there was no significant difference between the two groups in the complications including Postoperative fixation failure, Cutout, Implant breakage, Deep vein thrombosis, Wound infection, and rates of union. The presence of lateral femoral wall fracture did not seem to affect the rate of complications in our series. We believe that the importance of good fracture reduction and proper implant positioning may play a key role on achieving proper results, and the lateral wall fracture did not affect the rate of complications.

HHS and PPMS as a well-known scale for assessing the hip joint function. Deng et al. compared the quality of reduction and outcomes in intertrochanteric fracture treated with proximal femoral nail antirotation in elderly patients. They found there is no differences in HHS between incompetent and intact lateral wall groups [32]. Wang et al. compared the intramedullary nail in combination with reconstruction plate and intramedullary nail alone. In their results, the intramedullary nail with reconstruction plate had higher HHS and PPMS than without reconstruction plate [33]. However, Kim and his college hold the viewpoint that the displaced lateral femoral fracture fragments tend to reduce spontaneously without any additional fixation during the postoperative period in intertrochanteric fractures and lateral wall fractures fragment did need additional fixation after intramedullary nail [34]. The present study has not found significant difference in HHS and PPMS in intertrochanteric fracture with intact and incompetent lateral wall, which means that the long-term results of surgical treatment for intertrochanteric fractures has not influenced daily activities.

In this retrospective study, we found that the mean operative time, blood loss during surgery were significantly less in intact group's than in the incompetent group. It was especially difficult and time consuming to reduction, which needs to repeatedly require multiple anteroposterior and lateral images. This may increase the time of surgery in incomplete group.

We acknowledge some limitations of the current study. First, the retrospective study design might have resulted in a selection bias. Second, the relatively small sample size might have an impact on clinical outcomes. Therefore, our results should be confirmed in larger multicenter randomized controlled trials with a longer follow-up period.

## Conclusion

In addition to increased blood loss and operation time for intertrochanteric fractures with lateral wall fractures, there is no significant difference between functional outcomes and complication. We believe that intramedullary nail is enough to fix the intertrochanteric fracture with or without lateral wall fracture.

## Data Availability

The datasets used and/or analyzed during the current study are available from the corresponding author on reasonable request.

## Abbreviations

PFNA: Proximal femoral nail anti-rotation
BMI: Body mass index
ASA: American Society of Anesthesiologists
HHS: Harris Hip Score
PPMS: Parker − Palmer mobility score
